# Time-dependent changes in gene expression induced by secreted amyloid precursor protein-alpha in the rat hippocampus

**DOI:** 10.1186/1471-2164-14-376

**Published:** 2013-06-06

**Authors:** Margaret M Ryan, Gary P Morris, Bruce G Mockett, Katie Bourne, Wickliffe C Abraham, Warren P Tate, Joanna M Williams

**Affiliations:** 1 Brain Health Research Centre, University of Otago, PO Box 56, Dunedin New Zealand; 2 Department of Anatomy, Otago School of Medical Sciences, PO Box 913, Dunedin New Zealand; 3 Department of Biochemistry, Otago School of Medical Sciences, PO Box 56, Dunedin New Zealand; 4 Department of Psychology, University of Otago, PO Box 56, Dunedin New Zealand; 5 Neuroscience Department, Garvan Institute of Medical Research, Sydney, Australia

**Keywords:** Secreted amyloid precursor protein alpha, Hippocampus, Organotypic slice cultures, Microarray, Ingenuity pathway analysis, Neuroprotection, Immediate early genes, MicroRNA, NF-κB

## Abstract

**Background:**

Differential processing of the amyloid precursor protein liberates either amyloid-ß, a causative agent of Alzheimer’s disease, or secreted amyloid precursor protein-alpha (sAPPα), which promotes neuroprotection, neurotrophism, neurogenesis and synaptic plasticity. The underlying molecular mechanisms recruited by sAPPα that underpin these considerable cellular effects are not well elucidated. As these effects are enduring, we hypothesised that regulation of gene expression may be of importance and examined temporally specific gene networks and pathways induced by sAPPα in rat hippocampal organotypic slice cultures. Slices were exposed to 1 nM sAPPα or phosphate buffered saline for 15 min, 2 h or 24 h and sAPPα-associated gene expression profiles were produced for each time-point using Affymetrix Rat Gene 1.0 ST arrays (moderated *t*-test using Limma: p < 0.05, and fold change ± 1.15).

**Results:**

Treatment of organotypic hippocampal slice cultures with 1 nM sAPPα induced temporally distinct gene expression profiles, including mRNA and microRNA associated with Alzheimer’s disease. Having demonstrated that treatment with human recombinant sAPPα was protective against *N*-methyl d-aspartate-induced toxicity, we next explored the sAPPα-induced gene expression profiles. Ingenuity Pathway Analysis predicted that short-term exposure to sAPPα elicited a multi-level transcriptional response, including upregulation of immediate early gene transcription factors (AP-1, *Egr1*), modulation of the chromatin environment, and apparent activation of the constitutive transcription factors CREB and NF-κB. Importantly, dynamic regulation of NF-κB appears to be integral to the transcriptional response across all time-points. In contrast, medium and long exposure to sAPPα resulted in an overall downregulation of gene expression. While these results suggest commonality between sAPPα and our previously reported analysis of plasticity-related gene expression, we found little crossover between these datasets. The gene networks formed following medium and long exposure to sAPPα were associated with inflammatory response, apoptosis, neurogenesis and cell survival; functions likely to be the basis of the neuroprotective effects of sAPPα.

**Conclusions:**

Our results demonstrate that sAPPα rapidly and persistently regulates gene expression in rat hippocampus. This regulation is multi-level, temporally specific and is likely to underpin the neuroprotective effects of sAPPα.

## Background

Amyloid precursor protein (APP) is the parent molecule of the neurotoxic amyloid-β, implicated in the aetiology of Alzheimer’s disease. Amyloid-β is generated by sequential cleavage of APP by β and γ-secretases. However, the likely predominant route of APP processing is via α-secretase [1,2], which not only precludes amyloid-β production but also generates secreted amyloid precursor protein alpha (sAPPα) [3,4]. This molecule interacts with the β-secretase, BACE1, and directly inhibits amyloid-β production [5]. Furthermore, mounting evidence implicates sAPPα in a wide variety of neuronal processes. It has been shown to both protect against glutamate toxicity in rat hippocampal neurons *in vitro* and in rat models of traumatic brain injury *in vivo*[6,7] and promote neurite outgrowth *in vitro*[8,9]. Furthermore, sAPPα has recently been shown to not only direct human embryonic stem cells into neuronal precursor cells [10,11], but to increase proliferation of neural precursor cells from the rat hippocampus in vitro, and promote their proliferation in the mouse hippocampus *in vivo*[12]. Additionally, sAPPα has been shown to enhance synaptic plasticity and restore memory deficits in rats and mice *in vivo*[13-16]. While a definitive cognate receptor for sAPPα has not yet been identified, sAPPα is known to activate intracellular signalling cascades in both neurons and glia and enhance synaptic protein synthesis *in vitro*[17-21]. As the reported physiological effects of sAPPα, are dependent on rapid and persistent alterations in gene expression, it is also likely that regulation of transcription is integral to the function of sAPPα.

Recently, expression of the transcription factor *Egr1* was shown to mediate sAPPα stimulated axonal outgrowth of primary neurons from mice *in vitro*[22], and sAPPα has previously been shown to induce the expression of several neuroprotective genes in mouse organotypic hippocampal slices [23]. This observation is particularly important as the hippocampus is a region vulnerable to the early neurodegenerative changes observed in Alzheimer’s disease. Interestingly, however, sAPPα knock-in mice show significantly lower expression of the plasticity related genes *Arc*, *Egr2* and *Fos*. Furthermore, in general, the gene expression profiles of APP knockout and sAPPα knock-in mice do not differ substantially [24], questioning the role of the *N*-terminal region of APP in regulation of gene expression. In light of these somewhat conflicting data we sought to determine whether the application of sAPPα to organotypic hippocampal slice cultures for up to 24 h alters gene expression, and if so, whether the gene networks or biological pathways identified reflected the known physiological roles of sAPPα. We found that sAPPα rapidly enhances the transcription environment and alters subsequent gene expression in a manner that is likely to underpin the reported neuroprotective effects of sAPPα.

## Results

### sAPPα protects against *N*-methyl d-aspartate toxicity

We have previously shown that purified human recombinant sAPPα produced in our laboratory [25] regulates protein synthesis [17], synaptic plasticity and memory [16], as well as neurogenesis [10]. However, prior to determining the effects of sAPPα on gene expression, we sought to extend our assessment of the functionality of recombinant sAPPα. Accordingly, we tested whether sAPPα could protect against *N*-methyl-d-aspartate (NMDA)-induced excitotoxicity in hippocampal organotypic slice cultures.

**Figure 1:**
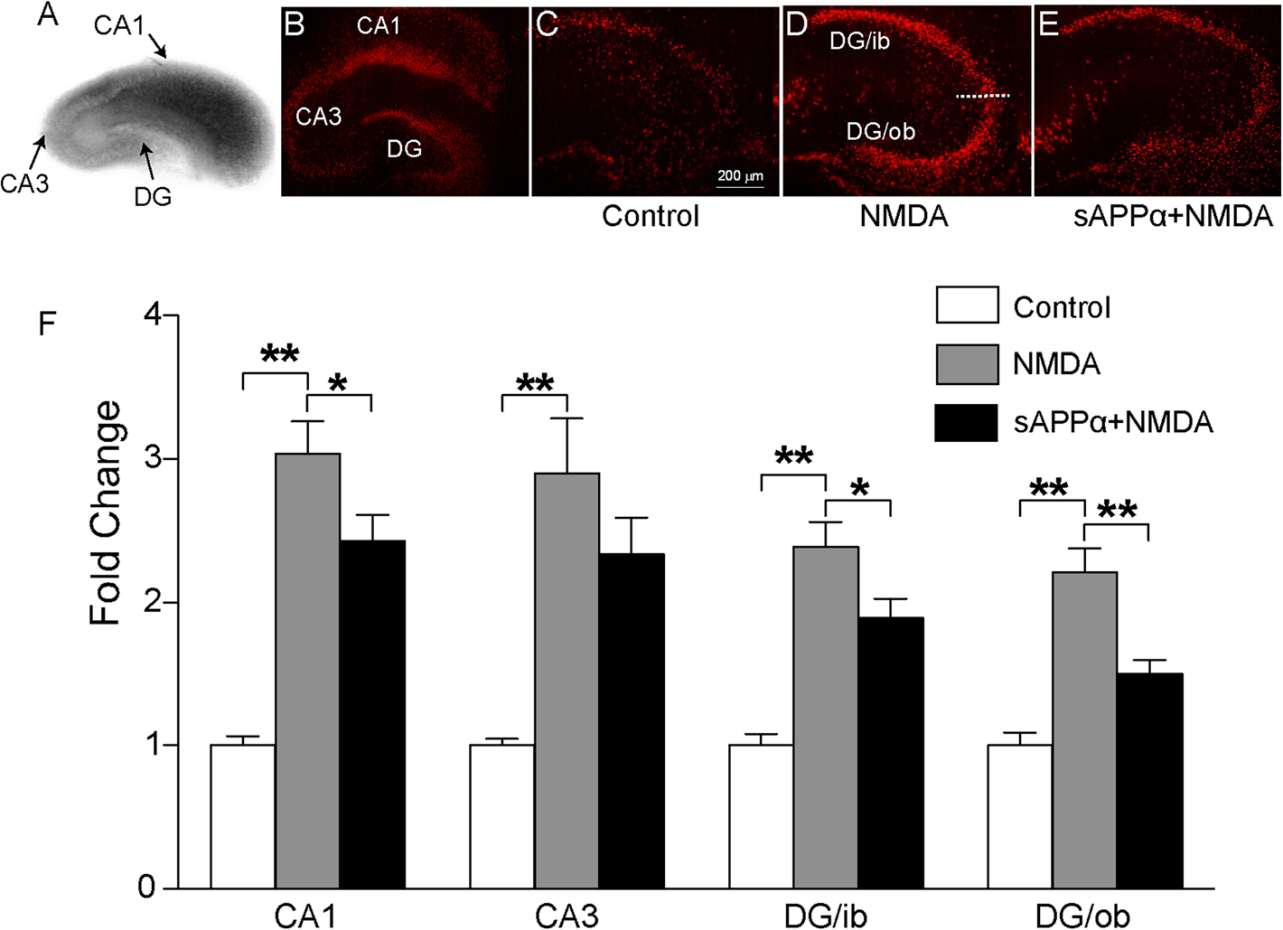
**sAPPα protects organotypic hippocampal slices from excitotoxic cell death.** (**A**) Representative brightfield image of a rat organotypic hippocampal slice. (**B**) Composite hippocampal slice showing high PI fluorescence, associated with total cell death in CA1 and CA3 and the dentate gyrus (DG) inner (ib) and outer blades (ob). Total cell death was induced by maintaining the slice at room temperature for 24 h in the absence of elevated CO_2_ levels. (**C**) Minimal PI fluorescence in the DG of a PBS-treated control slice. (**D**) Widespread PI fluorescence in the DG 48 h after NMDA challenge. (**E**) Reduced PI fluorescence in the DG following treatment with sAPPα and NMDA. (**F**) Regional analysis of PI fluorescence in organotypic hippocampal slices. Fold change: NMDA or NMDA + sAPPα PI fluorescence relative to PBS-treated control. NMDA treatment resulted in a >2 fold increase in PI fluorescence across all regions compared to PBS-control treated slices. Co-incubation with sAPPα and NMDA significantly reduced PI fluorescence in the CA1, DG/ib and DG/ob compared to NMDA alone. 1-way ANOVA with a Bonferroni post-hoc test: *p < 0.05, **p < 0.01, n = 4 animals, 19 slices/group.

Hippocampal organotypic slice cultures (representative slices, Figure 1A, B) were challenged with NMDA (30 μM, 30 min) and cell death was assessed by measurement of propidium iodide (PI) fluorescence 48 h later. In phosphate buffered saline (PBS)-treated control cultures, the tissue had low PI fluorescence across all hippocampal regions, indicating little cell death (Figure 1C). In contrast, NMDA treatment caused significantly higher levels of PI fluorescence in Cornu Ammonis area 1 and 3 (CA1, CA3) and the inner and outer blades of the dentate gyrus (DG) relative to the PBS-treated control cultures (*p* = 0.000001, 1-way ANOVA followed by a Bonferroni post-hoc test; Figure 1D, F), indicating induction of extensive cell death. Slices were incubated with 0.03, 0.1, 1, 10 nM sAPPα or PBS-control for 24 h prior to, and for 48 h following NMDA insult. Incubation with 1 nM sAPPα resulted in a significant reduction in PI fluorescence in CA1 (*p* = 0.033) and the inner (*p* = 0.034) and outer blades (*p* = 0.0001) of the DG compared with NMDA treatment alone (Figure 1E, F), indicating a neuroprotective effect. No significant effects were detected following incubation with the other sAPPα + NMDA concentrations but sAPPα exposure trended towards a U-shaped dose–response curve (see Additional file 1: Figure S1). Together, these findings indicate that 1 nM sAPPα increases cell survival following NMDA exposure. Although sAPPα has previously been shown to protect against glutamate toxicity [6], glutamate and NMDA may induce excitotoxicity by non-overlapping signaling pathways [26], and so this study extends the known neuroprotective effects of sAPPα and validates the activity of sAPPα in our model system.

### Regulation of gene expression by sAPPα

**Figure 2:**
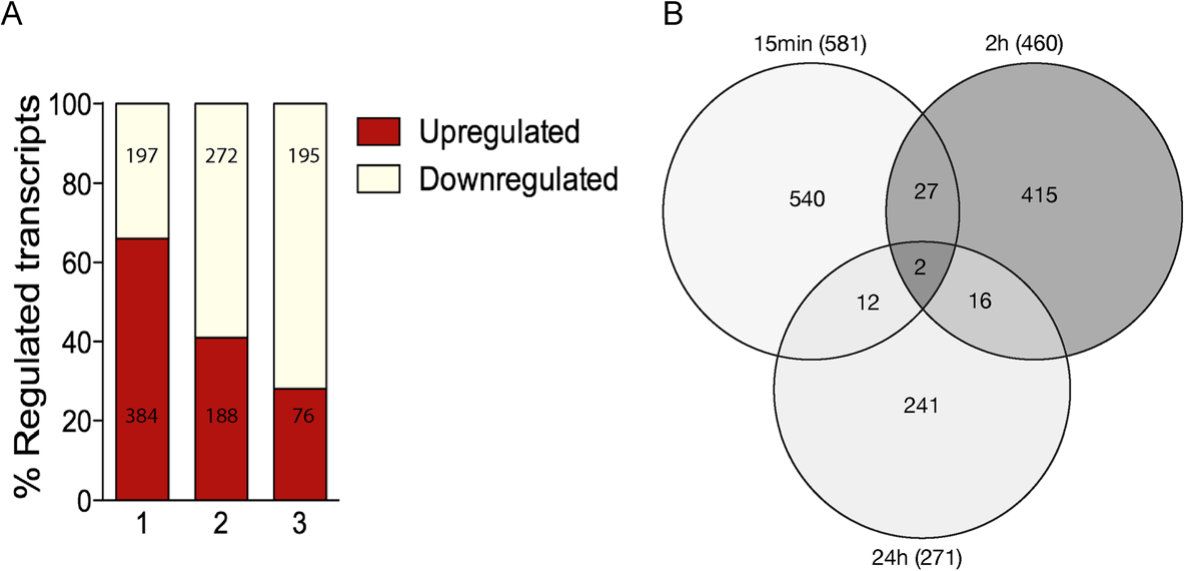
**sAPPα regulates gene expression in a temporally specific manner.** (**A**) Histogram showing that the majority of genes identified as differentially expressed following exposure of organotypic hippocampal slice cultures to sAPPα were upregulated at 15 min, while the majority were downregulated at 2 h and 24 h. Differentially expressed genes were identified using a dual selection criteria (±1.15 fold change; *p* < 0.05), see text for further details. (**B**) Venn diagram showing that the three datasets involved distinct gene sets.

**Figure 3:**
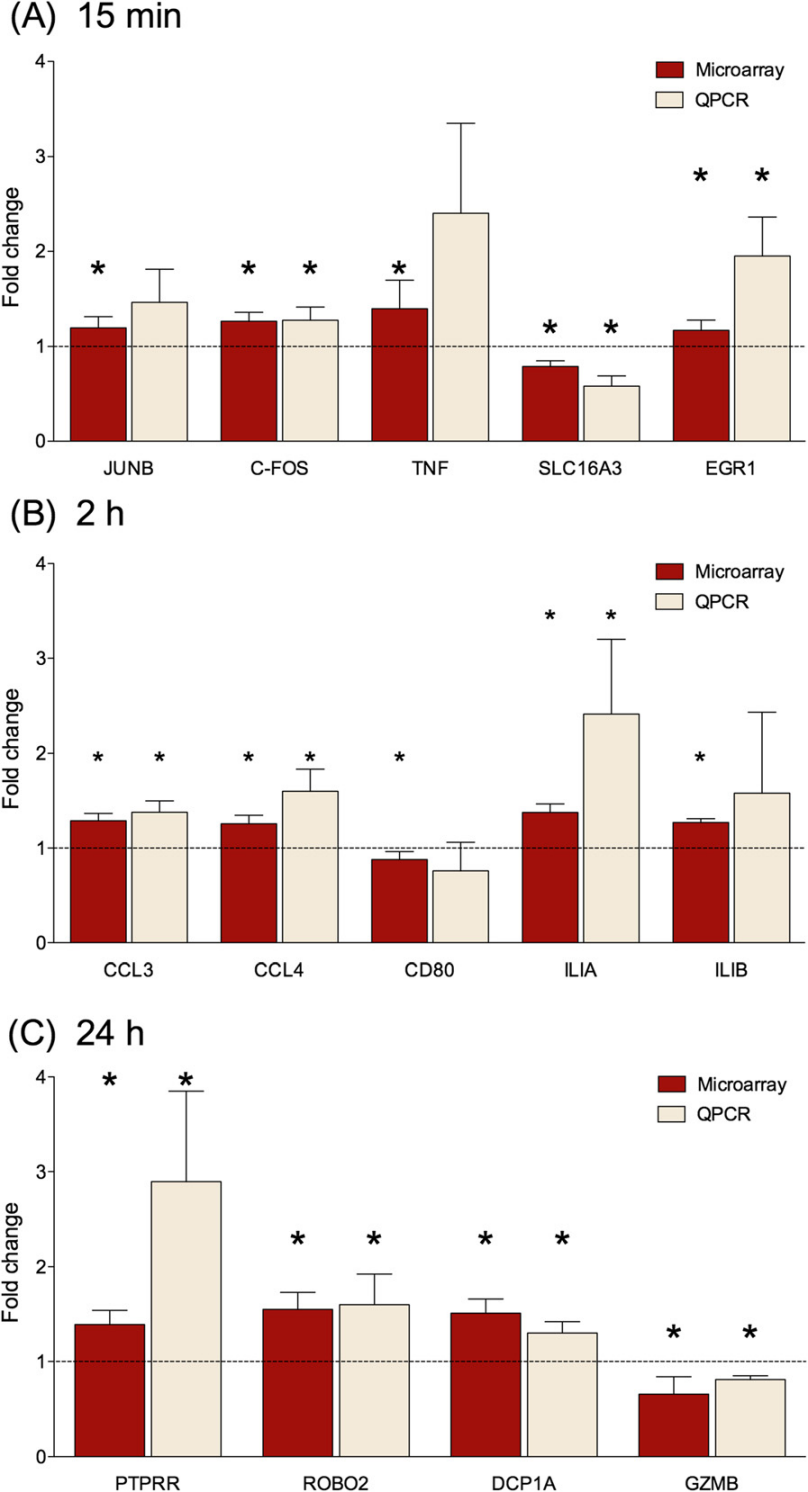
**Validation of selected differentially expressed genes by qPCR.** Microarray and corresponding qPCR results for (**A**) 15 min (**B**) 2 h (**C**) 24 h. Results are expressed as mean fold change +/− SEM and normalised to the housekeeping genes HPRT or PPIA using the 2^-∆∆^CT method; n = 5 per group. Significance was assessed using 1 tailed Student’s *t*-tests: * *p* < 0.05.

To investigate the temporal effects of sAPPα on the hippocampal transcriptome, hippocampal organotypic slice cultures were treated with 1 nM sAPPα (the optimally neuroprotective concentration) or PBS for either 15 min, 2 h or 24 h and expression profiling was performed with Affymetrix Rat Gene 1.0 ST arrays (n = 5 per condition). Data analysis, using dual selection criteria (moderated *t*-test, *p* < 0.05 (Limma) and fold change ± 1.15), identified temporally distinct sAPPα-induced patterns of expression (Figure 2A), a subset of which was confirmed by quantitative PCR (qPCR; Figure 3 and see Additional file 2: Tables S1-S4). The majority of genes were modestly changed (fold change: -2.9 to 3.2), an effect particular to neurobiological tissue [27] and consistent with previous studies [24,28-30]. Interestingly, two thirds (66%) of differentially expressed genes were upregulated in the 15 min dataset, while the majority of differentially expressed genes were downregulated at 2 h (59%) and 24 h (79%) (Figure 2A), suggesting that there is a relatively rapid onset homeostatic control of gene expression. Overall, these data confirm that sAPPα, the *N-*terminal region of APP, regulates gene expression in a time-dependent manner.

To explore further how the effects of sAPPα on gene expression changed with time, the expression profiles were examined using a Venn diagram. This analysis showed that the three datasets were largely distinct (Figure 2B; See Additional file 3: Table S5). Only two genes were in common across all three datasets and only one was annotated. Interestingly, this gene, acyl-protein thioesterase 2, (*Apt2*) showed a distinct temporal response to sAPPα treatment, decreasing in expression at 15 min (−1.25 fold), increasing at 2 h (+1.4 fold) and decreased by 24 h (−1.8 fold). Activation of APT2 results in depalmitoylation of substrate proteins, a process important in regulating subcellular localisation of plasticity related ion channels and GAP43 [31,32]. Thus, these data predict that regulation of *Apt2* contributes to the plasticity-related effects of sAPPα.

As sAPPα enhances synaptic plasticity and restores memory deficits [13-16], we next investigated whether there was a significant crossover between our sAPPα-regulated datasets and those regulated by synaptic plasticity. Previously, we identified distinct patterns of gene expression elicited by hippocampal long-term potentiation (LTP), a widely accepted model of memory mechanisms and synaptic plasticity [29,30]. Here, we show that there was little intersection between the genes within the datasets (Table 1). For example, there were only five rapidly responding genes in common across the early time-points analysed (Table 1) and the fold change in these overlapping genes was relatively curtailed in the sAPPα dataset (Table 1). Despite this, when we compared biological functions and pathways between the two data sets, we found that they contained overlapping functions related to *Gene Expression* (20 min LTP and 15 min sAPPα), *Development* (20 min LTP and 24 h sAPPα) and *Neurogenesis* (5 h LTP and 24 h sAPPα). This suggests that while there was little overlap in the actual genes regulated, there is overlap in some of the biological functions controlled by sAPPα and LTP.

**Table 1:** Intersection of sAPPα and plasticity-regulated datasets

**Gene symbol**	**Gene title**	**Fold change**	
		**15 min sAPPα**	**20 min LTP**
*Dusp1*	Dual specificity phosphatase 1	1.2	3.9
*Egr1*	Early growth response 1	1.2	4.2
*JunB*	Jun B proto-oncogene	1.2	6.6
*Rbp3*	retinol binding protein 3, interstitial	1.2	1.2
*Fos*	FBJ osteosarcoma oncogene	1.2	9.7
		**2 h sAPPα**	**5 h LTP**
*Grasp*	GRP1 (general receptor for phosphoinositides 1)-associated scaffold protein	1.2	1.3
		**24 h sAPPα**	**24 h LTP**
*Aox1*	Aldehyde oxidase 1	1.8	1.3
*Bhmt2*	Betaine-homocysteine methyltransferase 2	−1.4	−1.7
*Elov6*	ELOVL family member 6, elongation of long chain fatty acids	1.3	−1.3

As we found that sAPPα-induced a rapid upregulation followed by a generalised downregulation of gene expression, we hypothesised that this temporal pattern may be regulated by microRNA. A subset of probes on the Affymetrix Rat Gene 1.0 ST arrays represent stem loop microRNA sequences and therefore allow analysis of primary and precursor microRNA. We found evidence for regulation of 14 microRNA in response to sAPPα (Table 2), the majority of which were upregulated at 15 min, suggesting that the mature microRNA may contribute to the later observed downregulation of gene expression. In accordance with this, we found no evidence for regulation by microRNA within the 15 min dataset using the web application miRvestigator Framework [33]. However, analysis of the 2 h dataset identified miR-154 (*p* = 4.7e-04) as a candidate microRNA controlling the expression of a subset of mRNA (Table 3). As the miR-154 primary transcript was increased at 15 min (Table 2) this suggests a role for miR-154 in sAPPα-mediated gene expression, via mRNA degradation. Furthermore, miRvestigator predicted regulation by miR-201* (*p* = 2.4e-04) within the 24 h dataset (Table 3), although we found no evidence for altered levels of the primary microRNA transcript in our analyses.

**Table 2:** Temporally specific regulation of microRNA by sAPPα

**MicroRNA**	**Fold change**		
	**15 min**	**2 h**	**24 h**
mir-9	1.3	−1.2	-
mir-27	1.2	-	-
mir-29	1.3	-	-
mir-30	1.2	-	-
mir-130	1.2	-	-
mir-135	1.3	-	-
mir-154	1.2	-	-
mir-204	-	-	−1.7
mir-218	1.2	-	-
mir-219	-	1.2	-
mir-299	1.2	-	-
mir-505	-	1.2	-
mir-544	1.2	-	-
mir-592	1.2	-	-

**Table 3:** Predicted microRNA-regulated genes following miRvestigator Framework Analysis

**Predicted miR-154 regulated genes**	**Gene title**	**Transcript cluster ID**	**Fold change**	***P*. Value**
*Cks2*	CDC28 protein kinase regulatory subunit 2	10797559	−1.18	2.11E-02
*Crygd*	Crystallin, gamma D	10928549	−1.17	4.33E-02
*Dapp1*	Dual adaptor of phosphotyrosine and 3-phosphoinositides	10826985	−1.18	5.54E-03
*Hspa8*	Heat shock 70kDa protein 8	10826604	−1.18	6.75E-03
*Luzp1*	Leucine zipper protein 1	10880685	1.15	3.83E-02
*Olr1014*	Olfactory receptor 1014	10893397	−1.17	5.47E-03
*Olr1106*	Olfactory receptor 1106	10906853	−1.17	4.04E-02
*Olr1560*	Olfactory receptor 1560	10750649	−1.20	3.44E-02
*Olr1679*	Olfactory receptor 1679	10830769	−1.15	3.47E-03
*Olr75*	Olfactory receptor 75	10709401	−1.19	2.45E-02
*Olr862*	Olfactory receptor 862	10879329	1.22	1.45E-02
*Polr3G*	Polymerase (RNA) III (DNA directed) polypeptide G (32kD)	10718152	1.18	1.46E-02
*Rgd1309139*	Similar to CG5435-PA	10818370	−1.15	4.43E-02
*Loc303448*	Similar to glyceraldehyde-3-phosphate dehydrogenase	10715252	−1.15	3.41E-02
*Slc5A7*	Solute carrier family 5 (choline transporter), member 7	10921208	−1.19	2.23E-02
*St18*	Suppression of tumorigenicity 18	10875154	−1.21	1.98E-02
Predicted miR-201* regulated genes	**Gene title**	**Transcript cluster ID**	**Fold change**	***P*. value**
*Olr139*	Olfactory receptor 139	10709469	−1.43	2.61E-02
*Olr1532*	Olfactory receptor 1532	10750614	−1.72	4.48E-02
*Rpl36Al*	Ribosomal protein L36a-like	10829826	−1.36	4.34E-02
*Rplp2*	Ribosomal protein, large, P2	10762590	−1.43	3.70E-02
*Rgd1307947*	Similar to RIKEN cDNA C430008C19	10822064	−1.46	2.97E-02
*Wwc2*	WW and C2 domain containing 2	10788101	1.42	4.01E-02

As production of the Alzheimer’s disease-related amyloid-ß and sAPPα are mutually exclusive, we analysed how Alzheimer’s disease-associated genes were affected in our three data sets. To achieve this we compared our datasets with the Alzheimer gene database, AlzGene [34] and carried out an in-depth literature search. We identified a group of mRNA and microRNA previously associated with amyloid-ß induced toxicity (e.g. *Frp2* and *Ppif*), or implicated in Alzheimer’s disease processes, (miR-29 and miR-9) [35-47], (Table 4). As the expression of these genes was opposite to that observed in Alzheimer’s disease this analysis suggests that sAPPα may act, in part, through the same pathways as amyloid-ß.

**Table 4:** **sAPPα regulates genes linked to amyloid**-ß **toxicity and Alzheimer’s disease**

**Gene symbol**	**Gene title**	**Fold change**			**Functional effects**	**References**
		**15 min**	**2 h**	**24 h**		
*Fish*	Adapter protein TKS5	1.2	-	-	Mediates the neurotoxic effect of amyloid-β	[35]
*Frp2*	Formyl peptide receptor 2	-	-	−1.4	Activated by amyloid-β, may mediate inflammation seen in Alzheimer's disease	[36]
*Gapdh*	Glyceraldehyde-3-phosphate dehydrogenase	1.2	-	−1.5	May interact with neurodegenerative disease-associated proteins	[37]
*Ppif*	Peptidylprolyl isomerase F	-	-	−1.4	An absence of this gene is neuroprotective against amyloid-β induced cell death	[38]
*Tnf*	Tumour necrosis factor	1.4	-	-	Protects neurons against amyloid-β induced toxicity	[39]
*Ttr*	Transthyretin	-	−2.8	-	Binds amyloid-β and prevents fibril formation *in vitro*	[40]
*Ccl-3*	Chemokine (C-C motif) ligand 3	-	1.3	-	Protein levels are increased in Alzheimer's disease	[41]
*Fos*	FBJ murine osteosarcoma viral oncogene homolog	1.3	-	-	Gene expression is increased in Alzheimer's disease	[42]
*Ilib*	Interleulin 1-beta	-	1.3	-	Protein levels are increased in Alzheimer's disease	[43]
*MiR-9*	MicroRNA- 9	1.2	−1.2	-	Expression is reduced in Alzheimer's disease	[44]
*MiR-29*	MicroRNA-29	1.3	-	-	Regulator of BACE1 and decreased in Alzheimer's disease	[45]
*Otc*	Ornithine transcarbamylase	−1.2	-	-	Gene expression is increased in Alzheimer's disease	[46]
*Rgsl2*	Regulator of G-protein signaling like 2	-	1.2	-	Gene associated with late onset Alzheimer's disease	[47]

### sAPPα rapidly regulates transcriptional processes

**Figure 4:**
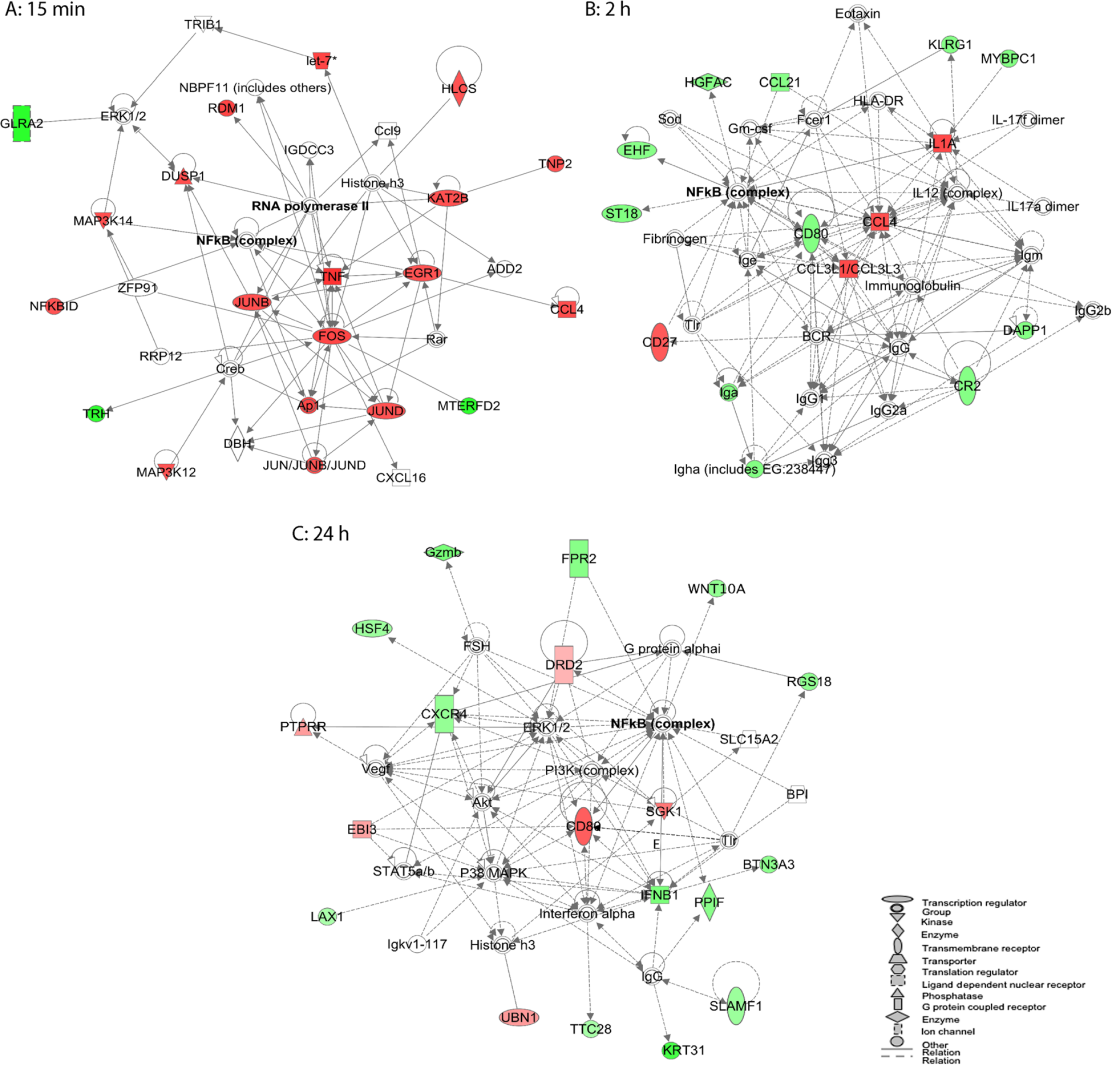
**IPA network analysis of temporally-specific sAPPα-regulated datasets.** (**A**) The highest scoring “direct” interaction network (Score = 34) derived following 15 min exposure to sAPPα. (**B**) The highest scoring “all interactions” network (Score = 26) derived following 2 h exposure to sAPPα. (**C**) The highest scoring “all interactions” network (Score = 35) derived following 24 h exposure to sAPPα. Red: upregulated genes. Green: downregulated genes. White open nodes: genes not identified as differentially expressed but predicted to interact with sAPPα-regulated genes by IPA. A solid line denotes a direct functional interaction of the products of the two genes. A dotted line denotes an indirect interaction. An arrow indicates that a gene product “acts on” a target.

**Figure 5:**
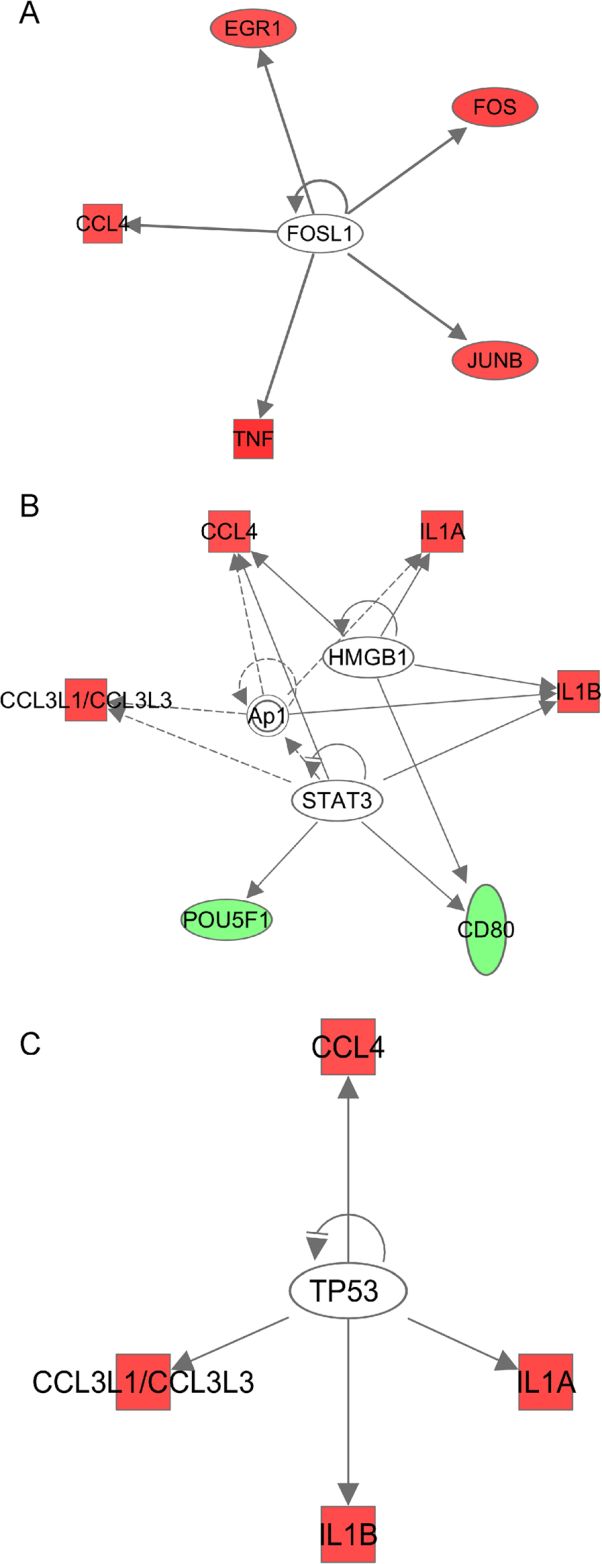
**IPA transcription factor analysis.** Each coloured node represents a gene; Red: upregulated genes. Green: downregulated genes. White open nodes represent Transcription factors. A solid line denotes a direct functional interaction of the transcription factor on the genes. A dotted line denotes an indirect interaction. (**A**) 15 min upstream analysis results (**B**) 2 h upstream analysis results (**C**) 24 h, upstream analysis results, up-regulated genes only.

To systematically investigate the functional significance of the genes rapidly regulated by sAPPα, we used the Ingenuity Pathway Analysis (IPA) software to identify gene networks from the 15 min dataset. *Gene expression* was identified as the most significant biological function of the highest scoring network, with the majority of the genes in this network upregulated (Figure 4A; score 34). Analysis of this network showed multiple mechanisms engaged by sAPPα to upregulate gene expression. This includes inducible transcription factors of the AP-1 complex [48] (FOS, JUNB and JUND) and the early growth response transcription factor, EGR1. Enhanced expression of *Fos* and *Egr1* was confirmed by qPCR (Figure 3; Additional file 2: Table S4.); furthermore, the upstream regulatory analysis tool within IPA predicted downregulation of a negative regulator of AP-1 activity *Fosl1/Fra1* (FOS-like antigen 1) [49,50], an effect likely to further amplify the sAPPα-induced transcriptional response (Figure 5A).

This network also predicts that sAPPα mediates transcription via activation of the plasticity-related transcription factors, cAMP response element binding protein (CREB) and nuclear factor kappa B (NF-κB), that form central hubs within the network. While their expression levels remain unchanged, this is highly consistent with their roles as constitutive transcription factors regulated by post-transcriptional modification. Indeed, this network includes *Dusp1* (+1.2 fold), a plasticity-related phosphatase and *Map3k14* (+1.2 fold), a kinase known to phosphorylate and activate NF-κB, as well as the NF-κB regulatory molecule, *Nf-κbid* (+1.2 fold) [51,52]. Together these data suggest that sAPPα treatment not only enhances the levels of transcription factors but also contributes to a dynamic interplay between a subset of protein kinases and phosphatases likely to maintain transcriptional activity.

Regulation of the chromatin environment is a third predicted mechanism by which sAPPα regulates expression of genes in this network. The network contains mediators of chromatin condensation (*Kat2b*: K(lysine) acetyltransferase; +1.2 fold) and histone biotinylation (*Hlcs*: holocarboxylase synthetase (biotin-(proprionyl-CoA-carboxylase (ATP-hydrolysing)) ligase; +1.2 fold) [53,54]. Upregulation of KAT2B is likely to result in reduced condensation of chromatin, occurring via acetylation of TNP2 (nuclear transition protein 2) a molecule also regulated within this network.

To further explore the 15 min dataset we used the functional annotation tool, DAVID. This highlighted *Cognition* and *Sensory perception* (Enrichment Score 2.2) as well as *Response to corticosterone* (Enrichment Scores 1.77, 1.34) as significantly enriched functions (Additional file 4: Table S6). Interestingly, there is an increase in basal glucocorticoid levels in Alzheimer’s disease, [55,56]. Thus sAPPα may regulate the expression of genes associated with this pathway.

### sAPPα-induced regulation of apoptosis and the inflammatory response

We next explored the gene networks affected by exposure to sAPPα for extended times. Following 2 h exposure to sAPPα, *Inflammatory Response* was the most significant biological function of the highest scoring network formed used IPA (Figure 4B; score 26). This network contains the proinflammatory cytokine *Il1a*, previously associated with Alzheimer’s disease, development and plasticity [57-61], immune associated genes (*Cd80*, *Cr2, Cd27*, *Dapp1*) and chemokines (*Ccl3, Ccl4, Ccl21*). These chemokines are involved in chemoattraction of immune cells to sites of tissue damage but have widespread non-immunological effects in the central nervous system, including regulation of neural cell proliferation, migration, survival and synaptic transmission [62,63]. That sAPPα treatment promotes regulation of chemokine responses is also supported from analysis of the 2 h data set with the functional annotation tool, DAVID (Additional file 4: Table S6).

Like the 15 min network, NF-κB forms a central hub in this network, however, many of the genes contributing to the hub, including *Ehf* (ets homologous factor), which promotes apoptosis [64], are downregulated. Interestingly, using the upstream regulatory element analysis tool to analyse the genes upregulated within the 2 h dataset, it is predicted that sAPPα induces inhibition of the transcription factor, tumour protein 53 (p53), which is also an important regulator of apoptosis, and is increased in Alzheimer’s disease [65,66] (Figure 5B). In addition, upstream regulatory element analysis using the entire 2 h dataset, predicts activation of the transcription factors AP-1, HMGB1 (high mobility group box protein) and STAT3 (signal transducer and activator of transcription 3) (Figure 5C) suggesting other mechanisms by which sAPPα coordinates this gene response.

### sAPPα-induced long-term regulation of neurogenic, inflammatory response and cell survival pathways

As the reported biological functions of sAPPα, such as neuroprotection and regulation of memory, are enduring physiological changes, we explored the gene expression response induced by a 24 h exposure to sAPPα. To identify the functional relationships within the 24 h differentially expressed gene list, we used the DAVID functional annotation tool. This analysis identified *Neurogenesis, Morphogenesis* and *Development* as important biological functions affected 24 h post-sAPPα treatment (Enrichment Scores 2.26-1.48) (Additional file 4: Table S6, See Additional file 5: Figure S2).

We extended analysis of this dataset by interpretation of the highest scoring network derived using IPA, (Figure 4C). This network was composed of genes with overlapping biological functions, including upregulated genes related to cell proliferation (*Cd80*, *Ubn1*, *Ptprr*; validated by qPCR; Figure 3; Additional file 2: Table S4), regulation of genes likely to promote cell survival (*Inkb1, Cd80, Sgk1, Fpr2, Cxcr4*) and inhibit apoptosis (*Gzmb*; validated by qPCR; Figure 3; *Cxcr4, Ifnb1, Sgk1*). As sAPPα has previously been shown to promote growth, survival and proliferation in neurons and stem cells [10,67,68] these findings supports a role for sAPPα-induced gene expression in mediating these events.

## Discussion

In order to better understand the role that regulation of gene expression may play in mediating the wide-ranging cellular effects of sAPPα, we performed a global transcriptome analysis in organotypic hippocampal slice cultures treated with 1 nM sAPPα for 15 min, 2 h or 24 h. We focused on the hippocampus as this region is essential to learning and memory and is especially vulnerable to degeneration in Alzheimer’s disease [69]. Our analysis demonstrated that sAPPα rapidly regulates gene expression by engaging multiple transcriptional regulatory mechanisms. This is evidenced by rapid and transient upregulation of inducible transcription factors (AP-1 complex, EGR1), temporally specific regulation of constitutively expressed transcription factors (NF-κB, CREB) and microRNA, as well as, modulation of the chromatin environment.

Network analysis showed that regulation of NF-κB is a consistent theme across all three time-points investigated. This supports previous work proposing that the neuroprotective effects of sAPPα are mediated through activation of NF-κB and subsequent enhanced activation of gene expression [70,71]. sAPPα-induced NF-κB activation is implicated in protecting neural cells from apoptosis, therefore our results suggest a role for specifically regulated genes in this process [72]. Interestingly, NF-κB has recently been intimately linked with amyloid-ß production [73,74] and sAPPα modulates APP processing [5]. Therefore, the control of amyloid-β production may be crucially dependent on the activation status of NF-κB, over which sAPPα has influence [70-72].

Increasing evidence points to the involvement of dysregulated microRNA in Alzheimer’s disease [75] and indeed an NF-κB-sensitive microRNA has been implicated in modulating the inflammatory circuit in Alzheimer’s disease [76]. Our results highlight a role for microRNA in sAPPα-induced regulation of gene expression. We found that a short-term exposure to sAPPα was associated with upregulation of a subset of microRNA. As medium to long-term sAPPα exposure resulted in a general decrease in gene expression, we propose that this may be mediated by microRNA. Interestingly, we reported a similar general effect on gene expression following the induction of LTP, which we have shown to be associated with altered levels of mature microRNA [30]. Our results also demonstrate that sAPPα induces a subset of plasticity-associated immediate early genes, however, we found little evidence that the sAPPα-induced gene response paralleled that of the LTP gene response. Therefore, post-translational modifications, and altered synaptic protein synthesis, are likely to mediate many of the effects of sAPPα on LTP [17,77,78].

It is of particular note that prolonged exposure to sAPPα resulted in persistent effects on gene expression that correlate closely with the documented neuroprotective and neurogenic roles of sAPPα. This interpretation is consistent with the only other microarray analysis of sAPPα-regulated gene expression in the hippocampus [23]. This study reported that treatment of mouse organotypic slices with 1 nM sAPPα for 24 h also resulted in regulation of several neuroprotective genes. Interestingly, there was little coherence between the genes identified in this study and our own. This may result from differences in the array type used (Affymetrix MG-U74Av2 vs. Rat Gene 1.0 ST), the number of annotated genes investigated (~9, 000 vs ~28, 000), the statistical analyses performed (Wilcoxon signed rank test vs. moderate paired *t*-test) or species (mouse vs. rat).

While there is a paucity of data estimating the physiological levels of sAPPα, it has been estimated to fall within the pM range within plasma and brain homogenates [79-81], however, it is difficult to extrapolate these concentrations to the *ex vivo* model used in this study. Our studies highlight that 1 nM sAPPα is sufficient to modulate gene expression in *ex vivo* models and emphasizes that both medium and longer-term exposure to sAPPα elicits an inflammatory and immune gene response, which likely provides a neuroprotective environment. This neuroprotective setting appears to be strengthened by a parallel downregulation of apoptotic pathways and increases in cell proliferation and survival. Furthermore, we found evidence for the regulation of genes associated with neurogenesis. Recent evidence has also linked sAPPα with the induction of neurogenesis in the mammalian brain [10-12,82]. As new brain cells have the capacity to integrate into previously established neural networks and contribute to hippocampal functioning [83], neurogenesis may also contribute to the sAPPα-induced neuroprotective and memory enhancing effects over the long-term.

## Conclusion

In summary, our analyses consolidate the concept that sAPPα regulates gene expression. We provide evidence that this occurs in a temporally specific manner, and occurs through providing an environment conducive for transcription that results in activation of immediate early gene transcription factors, known to mediate neuroprotection and proliferation [84,85], and regulation of microRNA. Furthermore, we demonstrate that gene networks constructed following medium and prolonged exposure to sAPPα reveal novel mechanisms likely to underpin and consolidate the neuroprotective stimulus induced by sAPPα in the hippocampus.

## Methods

### Organotypic hippocampal slice cultures

#### Preparation and maintenance

Organotypic hippocampal slice cultures were prepared from 7–10 day old Sprague Dawley rat pups of either sex according to the method of Stoppini *et al.*, [86]. The animals were deeply anaesthetized with ketamine (100 mg/kg, i.p.), using a protocol approved by the University of Otago Animal Ethics Committee to ensure minimal animal suffering. Brains were removed and placed in ice-cold filter-sterilized dissection media consisting of Minimum Essential Medium (MEM) containing Hank’s salts (95.5%, Gibco), penicillin-streptomycin solution (1%, Gibco), HEPES buffer solution (2.5%, Gibco) and 1M Tris–HCl (1%, Invitrogen, CA, USA). Hippocampi were then dissected free on an ice-cold glass plate and transverse slices cut at 400 μm on a Mcllwain tissue chopper (Mickle Laboratory Engineering, Surry, England). Separated slices were individually placed onto cell culture inserts (Millicell®, Millipore, MA, USA) held in 35 mm disposable petri dishes (Nunc, Denmark). To control for any gender differences, individual slices were randomised across all inserts. Slices were subsequently incubated in media containing MEM with Hank’s salts (50%), Hank’s balanced salt solution (25%, Gibco) and heat-inactivated horse serum (25%, Gibco) with 100 units of penicillin-streptomycin/ml and buffered to pH 7.2 with 1 M HEPES solution for three days at 37°C in a humidified incubator containing 5% CO_2_, and subsequently at 34°C. Culture medium was changed after the initial 24 h, and then every 3 days.

#### Experimental manipulation

Slices were allocated to experimental groups after 10–11 days *in vitro* (DIV). Neuroprotection assays and gene expression experiments contained slices from 4 animals, with 4–5 slices on each insert. All treatments were conducted in culture medium in which the horse serum was replaced with the same volume of MEM. Recombinant human sAPPα was produced using human embryonic kidney-293 cells in which the sAPPα gene fragment was stably integrated [23].

#### Neuroprotection assay

Organotypic hippocampal slices were pre-treated with sAPPα (0.03-10 nM) or PBS for 24 h before treatment with NMDA (30 μM, 30 min) to induce partial, hippocampal-wide neuronal cell death. sAPPα was removed during the NMDA challenge, but subsequently reintroduced at the same concentration for a further 48 h.

#### Fluorescence imaging

Levels of cell death were determined 48 h after NMDA treatment using fluorescence imaging. Dead cells were labelled using the fluorescent dye propidium iodide (PI, ex. 536 nm, em. 617 nm). Emitted fluorescence was imaged with a Zeiss Axio Scope A1 microscope fitted with an LED illumination system and fluorescence filter cube (LED module ex. 540–580 nm; Zeiss filter set 43, ex. 545 nm- excitation, 605 nm-emission). Images were acquired with a Scion Corporation (Frederick, MD, USA) 12-bit colour camera (model CFW-1612C) and Scion Corporation VisiCapture image acquisition software. This software enabled us to reduce electronic and background image noise by collecting images that were the average of the previous five frames. For the purposes of analysis, each cultured slice was divided up into four principal areas CA1, CA3, inner blade of the dentate gurus (DG/ib), and outer blade of the dentate gyrus (DG/ob).

#### Image analysis

Cell death was quantified by measuring the average pixel intensity within a manually-selected region-of-interest (ROI) using ImageJ software (http://rsbweb.nih.gov/ij/download.html). For each image, the cell body layer was identified and manually selected as a ROI from which the average pixel intensity was calculated. The positioning of each ROI was dependent on the area to be analysed; CA1: identified as the cell body layer immediately above the DG; CA3: identified as the layer continuous with, and extending beyond, CA1 and ending before it entered the hilus of the DG. DG: divided into inner and outer blades with an approximate mid-point selected to demarcate the segments (Figure 1). The ROI size for each of the four areas was kept consistent between images.

#### Data analysis

For assessment of regional sAPPα effects, raw PI intensity values in the sAPPα groups were compared across the control and NMDA group values using a 1-way ANOVA with a post-hoc Bonferroni correction. Significance was set at *p* < 0.05.

### Transcriptome analysis

Organotypic hippocampal slices were incubated with media containing 1 nM sAPPα or PBS for 15 min, 2 h or 24 h (n = 5 samples per treatment group, with each sample consisting of 5 randomly selected slices from 4 animals), rinsed with PBS, fixed in 70%(v/v) ETOH and snap frozen. Total RNA was prepared from organotypic hippocampal slices following tissue grinding on dry ice and shearing through a 22G × 1" Ultra Thin Wall Needle (Terumo®, Japan), using the NORGEN Total RNA Purification Kit (Norgen Biotek Corporation, Canada), plus DNAse I treatment step (Qiagen, Germany). RNA sample concentrations and integrity were determined using spectrophotometry (Nanodrop 1000; Thermo Scientific, USA) and a Bioanalyzer, using an RNA 6000 Nano Labchip (Bioanalyzer 2100; Agilent Techologies, USA). Only samples with an average RNA integrity number >8 were used [87,88]. Array hybridizations were carried out at the Otago Genomics Facility (University of Otago), where RNA samples were biotin-labeled and hybridized to Affymetrix Rat Gene 1.0 ST arrays. These arrays cover 27342 annotated genes represented by ~26 probes spread across the full-length of each gene. A subset of microRNA are represented on the Rat GeneChip. The microRNA specific probes align to the stem loop sequences with the potential to identify both the primary and precursor forms of the transcripts.

### Microarray data analysis

The Robust Multichip Average (RMA) package was used to normalize the data derived from treated and control samples (Sketch expression consul, Affymetrix, USA). In order to produce an inclusive list of sAPPα related genes and microRNA suitable for network analysis, differentially expressed genes were identified using two selection criteria: a threshold fold change cutoff (± 1.15) and a moderated *t*-test with a significance criterion of *p* < 0.05 [29]. The t-statistic was generated using the Limma package, which utilizes a standard error moderated across all genes using a simple Bayesian model and produces *p*-values with greater degrees of freedom and hence greater reliability [89]. More stringent selection criteria were not used in this study to avoid the risk of Type 2 error and unnecessarily limit the datasets for network analysis [90,91].

To investigate potential microRNA likely to regulate the observed datasets, we used the web application miRvestigator Framework (http://mirvestigator.systemsbiology.net/) that identifies microRNA responsible for co-regulated gene expression patterns [33,92].

### Identification of biologically relevant networks and biological functions

Ingenuity pathway analysis, version 9 (IPA) (Ingenuity® Systems, http://www.ingenuity.com) was used to investigate interaction-based relationships between the genes and proteins encoded by the sAPPα-regulated gene expression sets. The gene sets, containing Affymetrix identifiers and corresponding expression values (*p* < 0.05), were submitted for analysis and gene networks were produced. Each network contains up to 35 genes and has an associated score derived from a *p*-value, indicating the expected likelihood of the genes being present in a network compared to that expected by chance. Scores of two or above have at least a 99% likelihood of not being generated by chance.

As the use of multiple tools is highly recommended for functional analysis of microarray data [93], we also applied an alternative analysis approach, Functional Annotation Clustering (DAVID [93]) to analyse the differentially expressed genes. In contrast to IPA, which is an interaction-based analysis, DAVID categorises the genes into groups based on Gene Ontology terms, and then displays similar annotations together. The total set of genes on the appropriate microarray was used as the background. The annotation terms are clustered based on the share of common genes and an EASE score, a modified Fisher exact p-value, is produced for each annotation term. Usually a *p*-value equal or smaller than 0.05 is considered strongly enriched in the annotation categories [93]. Next, the annotation terms were clustered based on similar annotation terms and genes. The overall enrichment score of each cluster was based on the EASE scores of each annotation term. Clusters with a minimum *enrichment score* of 1.3 (equivalent to a *p*-value of 0.05) were deemed significant.

### Real-time quantitative PCR

Selected biologically relevant differentially expressed genes were analysed by quantitative PCR (qPCR). Extracted RNA was reverse-transcribed to first strand cDNA using Superscript III (Invitrogen, CA, USA). Sixteen primer pairs were designed using Primer 3 (http://primer3.wi.mit.edu/) and obtained from Integrated DNA Technologies, (USA). Primer sequences are described in Additional file 6: Table S7. qPCR was performed using SYBR green mastermix (Roche, Switzerland) on a Roche Lightcycler 480. The qPCR validation included animals from the same RNA used in the microarray analysis. Results were normalised to the housekeeping genes Hypoxanthine phosphoribosyltransferase 1 (*Hprt*) and Peptidylprolyl isomerase A (*Ppia*) using the 2^-∆∆^CT method [94]. These genes have consistently remained stable across numerous studies in the hippocampus eg [95,96]. Significance was assessed using Student’s *t*-tests with the criterion set at *p* < 0.05 and 1.2 fold change.

### Availability of supporting data

The data sets supporting the results of this article will be available in the ArrayExpress repository, accession number pending.

## Competing interests

The authors declare no competing interests.

## Authors’ contributions

MMR and GPM were the major contributors to the experimental aspects of the study. MMR conceived and designed the study, carried out two of the experimental array studies and validations, the statistical and data analysis and interpretation, and drafted the manuscript. GPM helped design the study, carried out one array study and validation, the corresponding data analysis and interpretation, and drafted the manuscript. BGM prepared the hippocampal organotypic slice cultures, and validated the neuroprotective function of sAPPα against excitotoxicity. KB developed the laboratory-based production and purification of sAPPα in human cells. WCA contributed to the design of the study and critically revised the manuscript. WPT and JMW were equal, complementary contributors to the overall leadership of the project. WPT conceived, participated in the design and co-ordinated the experimental aspects of the study, and helped draft the manuscript. JMW conceived and participated in the design and co-ordination of the study, undertook critical data analysis and interpretation, and drafted the manuscript. All authors read and approved the final manuscript.

## Supplementary Material

Additional file 1: Figure S1 Neuroprotective effects of sAPPα are concentration dependent. Change in cell death in DG/ob following treatment of sAPPα (0.03-10 nM) relative to control/NMDA treatment. *:p < 0.05, n = 4 animals, 19 slices/group. sAPPα neuroprotection followed a U-shaped dose–response curve, with increasing concentrations of sAPPα (to a maximum of 1 nM) leading to a significant neuroprotective effect, which then decreased with increasing concentrations of sAPPα. This agrees with our previous study [16], where we observed a dose dependent facilitation of *in vivo* LTP by sAPPα, with little effects on LTP at low concentrations, facilitation at mid-range concentrations and significant inhibition at high concentrations. This may reflect the occupancy/binding properties of the receptor of sAPPα, as yet unidentified [12,97]. No significant effects were detected following incubation with the other sAPPα + NMDA concentrations. Click here for file

Additional file 2: Tables S1-S4 Significantly expressed transcripts clusters following 15 min (**Table S1**), 2 h (**Table S2**) and 24 h (**Table S3**) sAPPα exposure. **Table S4:** Validation of selected differentially expressed genes by qPCR including associated *p*-values. Entries without gene symbols and titles have no annotations associated with these probe ids. Click here for file

Additional file 3: Table S5 Expression of sAPPα related genes across time. Overlapping temporal gene expression lists from Venn diagram (Figure 2B). NC: no change in gene expression at this time. Click here for file

Additional file 4: Table S6 DAVID functional annotation clustering of sAPPα-regulated genes. Click here for file

Additional file 5: Figure S2 Genes with annotations related to neurogenesis and development. Associations between the 24 h gene list and their annotations following DAVID functional analysis. The region in green illustrates that all annotations are common across the cluster. Enrichment score 2.35. Click here for file

Additional file 6: Table S7 List of primers used for real-time qPCR. Click here for file
