# Supplementary figures and images for: Time-dependent changes in gene expression induced by secreted amyloid precursor protein-alpha in the rat hippocampus
Source: BMC Genomics. 2013 Jun 6;14:376. doi: 10.1186/1471-2164-14-376 (PMC3691674; doi:10.1186/1471-2164-14-376)

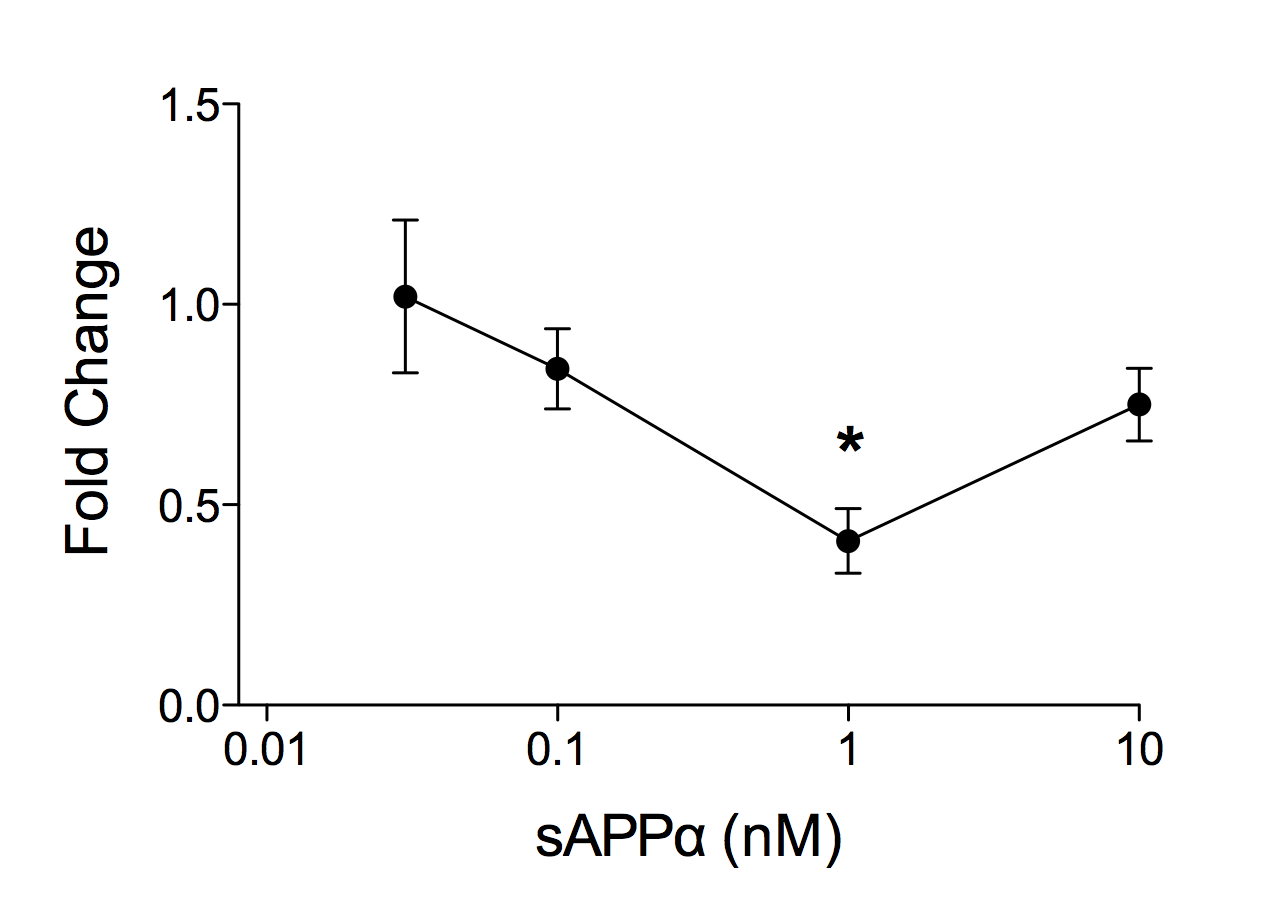

Supplement: Additional file 1: Figure S1 — Neuroprotective effects of sAPPα are concentration dependent. Change in cell death in DG/ob following treatment of sAPPα (0.03-10 nM) relative to control/NMDA treatment. *:p < 0.05, n = 4 animals, 19 slices/group. sAPPα neuroprotection followed a U-shaped dose–response curve, with increasing concentrations of sAPPα (to a maximum of 1 nM) leading to a significant neuroprotective effect, which then decreased with increasing concentrations of sAPPα. This agrees with our previous study [16], where we observed a dose dependent facilitation of in vivo LTP by sAPPα, with little effects on LTP at low concentrations, facilitation at mid-range concentrations and significant inhibition at high concentrations. This may reflect the occupancy/binding properties of the receptor of sAPPα, as yet unidentified [12,97]. No significant effects were detected following incubation with the other sAPPα + NMDA concentrations. [file 1471-2164-14-376-S1.tiff]

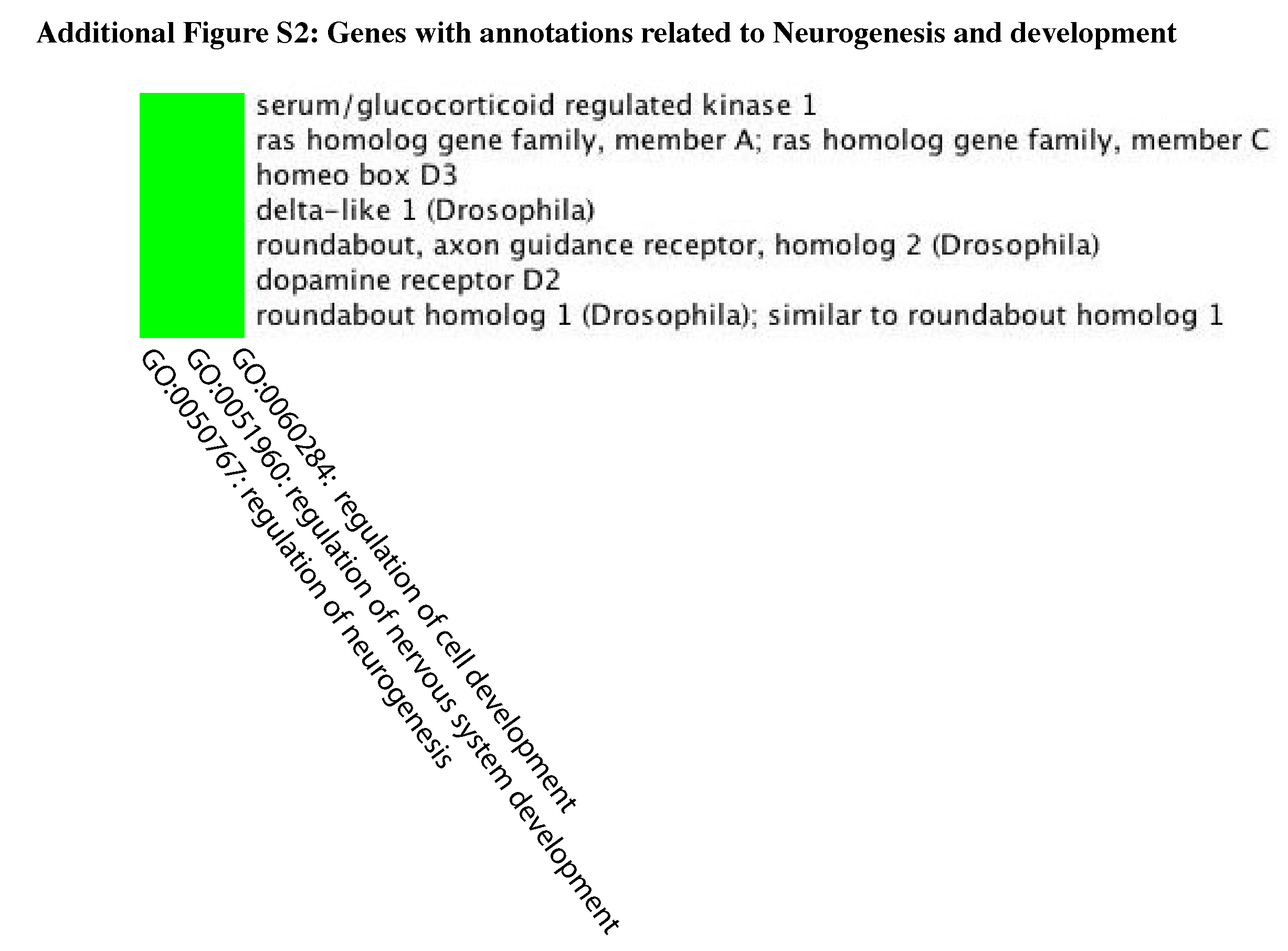

Supplement: Additional file 5: Figure S2 — Genes with annotations related to neurogenesis and development. Associations between the 24 h gene list and their annotations following DAVID functional analysis. The region in green illustrates that all annotations are common across the cluster. Enrichment score 2.35. [file 1471-2164-14-376-S5.tiff]
