# Supplementary material for: Time-dependent changes in gene expression induced by secreted amyloid precursor protein-alpha in the rat hippocampus
Source: BMC Genomics. 2013 Jun 6;14:376. doi: 10.1186/1471-2164-14-376 (PMC3691674; doi:10.1186/1471-2164-14-376)
Supplement: Additional file 6: Table S7 — List of primers used for real-time qPCR. [file 1471-2164-14-376-S6.docx]

**Table S7: List of primers used for real-time qPCR**

| **Gene Symbol** | **sense primer** | **anti-sense primer** |
| --- | --- | --- |
| *Hprt* | TGACACTGGTAAAACAATGCA | GGTCCTTTTCACCAGCAAGCT |
| *Ppia* | TGGCAAATGCTGGACCAAAC | TTCTTGCTGGTCTTGCCATTC |
| *Fos* | AGAAGGGGCAAAGTAGAGCAG | GCAGCCATCTTATTCCTTTCC |
| *Junb* | CGCTCAACCTGGCAGATCCT | CGCCTGTGTCTGATCCCTGA |
| *Slc16a3* | TCAATGGCTTCACTGACCTG | AGCCATGAGCACCTCAAACT |
| *Egr1* | GGGAGCCGAGCGAACAA | CGTTATTCAGAGCGATGTCAGAA |
| *Tnf* | CCCCAATCTGTGTCCTTCTAA | TACTTCAGCGTCTCGTGTGTT |
| *Ccl3* | GGCAGCGAGTACCAGTCCCT | AGGGCAGCGGTGGAGACCTT |
| *Ccl4* | TATGAGACCAGCAGCCTTTGC | GCACAGATTTGCCTGCCTTT |
| *Il1a* | AAGACAAGCCTGTGTTGCTGAAGG | TCCCAGAAGAAAATGAGGTCGGTC |
| *Il1b* | AATGACCTGTTCTTTGAGGCTGAC | CGAGATGCTGCTGTGAGATTTGAAG |
| *Cd80* | TGAGGCAAGCAGAGAAACAA | GGCAAATGGCTACCTTCAAA |
| *Ptprr* | TGTGGTGACACTGCAAATG | AAACAACTCAATCTGGCTCTTC |
| *Dcp1a* | CGCAGCCTCCCTGAGCAACA | CTGCGATCGGGATGGCACTGG |
| *Robo2* | GCCAGTCAACAACAGCAATAG | CTGGAGGCAGCACATCTC |
| *Gzmb* | GCCCACAACATCAAAGAAC | GGCCTTACTCTTCAGCTTTAAC |
